# Investigating the Recycling Potential of Glass Based Dye-Sensitized Solar Cells—Melting Experiment

**DOI:** 10.3390/ma14216622

**Published:** 2021-11-03

**Authors:** Fabian Schoden, Anna Katharina Schnatmann, Emma Davies, Dirk Diederich, Jan Lukas Storck, Dörthe Knefelkamp, Tomasz Blachowicz, Eva Schwenzfeier-Hellkamp

**Affiliations:** 1 Institute for Technical Energy Systems (ITES), Bielefeld University of Applied Sciences, 33619 Bielefeld, Germany; anna_katharina.schnatmann@fh-bielefeld.de (A.K.S.); emma.davies@fh-bielefeld.de (E.D.); jan_lukas.storck@fh-bielefeld.de (J.L.S.); doerthe.knefelkamp@fh-bielefeld.de (D.K.); eva.schwenzfeier-hellkamp@fh-bielefeld.de (E.S.-H.); 2 Institut für Glas- und Rohstofftechnologie GmbH, 37079 Göttingen, Germany; d.diederich@igrgmbh.de; 3 Institute of Physics—Center for Science and Education, Silesian University of Technology, 44100 Gliwice, Poland; tomasz.blachowicz@polsl.pl

**Keywords:** recycling, circular economy, dye-sensitized solar cell, glass recycling, ICP-OES, SEM-EDX, melting experiment

## Abstract

The effects of climate change are becoming increasingly clear, and the urgency of solving the energy and resource crisis has been recognized by politicians and society. One of the most important solutions is sustainable energy technologies. The problem with the state of the art, however, is that production is energy-intensive and non-recyclable waste remains after the useful life. For monocrystalline photovoltaics, for example, there are recycling processes for glass and aluminum, but these must rather be described as downcycling. The semiconductor material is not recycled at all. Another promising technology for sustainable energy generation is dye-sensitized solar cells (DSSCs). Although efficiency and long-term stability still need to be improved, the technology has high potential to complement the state of the art. DSSCs have comparatively low production costs and can be manufactured without toxic components. In this work, we present the world’ s first experiment to test the recycling potential of non-toxic glass-based DSSCs in a melting test. The glass constituents were analyzed by optical emission spectrometry with inductively coupled plasma (ICP-OES), and the surface was examined by scanning electron microscopy energy dispersive X-ray (SEM-EDX). The glass was melted in a furnace and compared to a standard glass recycling process. The results show that the described DSSCs are suitable for glass recycling and thus can potentially circulate in a circular economy without a downcycling process. However, material properties such as chemical resistance, transparency or viscosity are not investigated in this work and need further research.

## 1. Introduction

An important pillar against climate change is the transformation of the energy sector to 100% renewable energy [1]. However, the energy sector is not the only contributor to climate change. Another critical problem is the consumption and depletion of resources and our linear economy, which is a cause of extensive carbon emissions [2,3]. The concept of how the linear economy works is often described as “take, make, waste” [4]. Resources are mined or extracted, then processed into products that are sold, used and usually disposed of after a few weeks or months [5].

Therefore, the need for sustainable and recyclable renewable energy systems is becoming increasingly important [6]. Technologies are needed that not only harness renewable energy but that can also be repaired, remanufactured and recycled [3]. One technology that has great potential in this area is dye-sensitized solar cells (DSSCs) [7]. They can be made from non-toxic material, and the production process is less energy-consuming compared to silicon-based photovoltaics (c-Si PV) [8,9,10,11]. They can also be used in areas with low light intensity, such as indoors or in the morning and evening hours [12]. This means they are well suited to complement existing renewable energy technologies and can be integrated into windows, facades or cars, even as transparent versions, and into Internet of Things devices [13,14]. In addition, DSSCs are easy to manufacture and insensitive to impurities, which facilitates industrial scale-up and enables manufacturing in rural areas [6,15].

The operating principle of a DSSC can briefly be described as follows: DSSCs consist of two glass plates coated with fluorine-doped tin oxide (FTO). The FTO layer makes one side of the glass plate conductive. The front electrode is additionally coated with a semiconductor, typically TiO_2_. A dye can be incorporated into the porous TiO_2_ layer. The counter electrode is coated with graphite or platinum in addition to the FTO layer. The front and counter electrodes are connected by an electrolyte. When light excites the dye, electrons are released from the dye and transported through the TiO_2_ layer into an external circuit where the current can be used. The electrons enter the DSSC through the counter electrode and recombine with the acceptors of the electrolyte, reducing the electrolyte, which eventually leads to the reduction of the dye cation. The role of the graphite or platinum layer on the counter-electrode is that of a catalyst, allowing the electrons to make their way through the electrolyte to the TiO_2_ layer and complete the circuit [16,17,18].

So far, DSSCs are not ready for mass production, and industrial scale-up depends on further improvement of DSSC efficiency and long-term stability. The highest efficiencies for DSSCs are achieved by using toxic or scarce materials such as silver, cobalt, platinum and ruthenium dyes [13,19]. This type of DSSC achieves efficiencies of over 14% in laboratory tests [15]. In ambient light, Freitag et al. achieved a power conversion efficiency (PCE) of 28.9% [20]. DSSCs, based on non-toxic and abundant components, usually use plant dyes such as carotenoids, chlorophylls or anthocyanins [21,22]. The efficiency of such DSSCs is usually less than 1% [23,24,25]. Innovations in DSSC research are also likely to rely on organic dyes, not just natural dyes. However, organic dyes can be toxic and thus dangerous for the environment [26,27]. This could make a recycling process more complicated. Some natural dyes require solvents and energy for the purification process, similar to ruthenium dyes [28]. Organic or natural dyes for DSSC applications should be developed following the twelve principles of Green Chemistry [13,29].

Long-term stability can be improved by using gel or solid-state electrolytes [10,30,31]. However, long-term stability research is often avoided because time-consuming tests are required to evaluate stability, and this creates a conflict with respect to rapid publication [32]. Gel electrolytes can improve long-term stability, and the DSSCs are stable for at least 140 days [33]. Plastics such as polyacrylates or polyacrylonitrile are used in gel electrolytes and could hinder the recycling process due to their poor biodegradability [34,35]. When these plastics enter the environment and become microplastics, they can enter the food chain and harm organisms [36].

To improve energy conversion and reduce cost, quantum dots are used to fabricate quantum dot sensitized solar cells (QDSC) [37]. These QDSCs could potentially be subjected to a similar recycling process as glass-based DSSCs. However, the basic elements of the quantum dots are cadmium (Cd), lead (Pb) or other heavy metal compounds, which are themselves toxic [38,39].

For DSSCs to be competitive on the mass market, they need to have a lifetime of up to 5 years for textiles or similar wearable technologies [40]. For applications in or around the building, a lifetime of about 25 years must be achieved to compete with c-Si PV [41].

In 2019, the global DSSC market size was USD 90.5 million, and the compound annual growth rate for DSSCs from 2020 to 2027 is estimated at 12.4% [7]. DSSCs are a growing market, and according to several studies, the critical environmental factors of DSSCs are [13,15,41,42,43]:

- The use of critical raw materials such as platinum and cobalt.
- The drop in performance due to the instability of the electrolyte.
- Uncertain waste management.
- The high energy requirements in the production of transparent conductive oxide (TCO) glass.

The last point, the high energy requirement for the production of TCO glass, results from the life cycle analyses (LCA). In an LCA, the environmental impact of a product or material is assessed. In these LCAs, the life cycle of the glass of the DSSC was considered from cradle to grave [13,41,42]. Cradle-to-grave is the scope of LCA in this case, and environmental impacts are considered from resource extraction to disposal of the DSSC. If a cradle-to-cradle perspective were applied, i.e., the material is reused and recycled, the impact, especially the energy demand, would be significantly reduced [44].

Therefore, in this experiment, we wanted to investigate the suitability of non-toxic DSSCs with natural dyes for glass recycling. In the absence of experiments with practical approaches to glass recycling and DSSCs, we conducted a melting experiment to investigate possible solutions to close the loop. As part of the concept of a circular economy, material cycles must be freed of toxic components. This means either recycling concepts must be developed to separate toxic substances from the material loops or holistic design principles must be applied. With the help of the circular economy, products are manufactured that do not contain toxic substances and can therefore be recycled and reprocessed more easily. This also eliminates the need for complicated separation processes for toxic elements in material loops [3,4].

## 2. Materials and Methods

The glasses for the construction of DSSCs were purchased from Man Solar B. V. (Petten, The Netherlands). All glasses were coated on one side with FTO for conductivity. The front electrode was additionally coated with TiO_2_ as semiconductor. Glasses from different batches were investigated. Older glasses from 2018 were thin (40 mm × 20 mm × 2 mm, 4 g) and newer glasses from 2020 (40 mm × 20 mm × 3 mm, 6 g) were thick. From now on, the thin glasses will be referred to as samples t1A and the thick glasses as t1B. In order to perform this experiment within the framework of the circular economy school of thought, DSSCs from old experiments with and without gel polymer electrolytes were used for the melting experiment [45].

### 2.1. Investigating Glass Surface SEM-EDX

Scanning electron microscopy energy dispersive X-ray (SEM-EDX) measurement was performed to investigate the surface of the glasses (Carl Zeiss EVO MA10, Carl Zeiss AG, Oberkochen, Germany). In this method, a material is bombarded with electrons. If such an electron hits electrons in deeper spheres of the bombarded sample, it is knocked out of this atom. A vacancy in a lower atomic orbital causes an electron at a higher energy level to jump to that lower energy level. In doing so, it emits characteristic X-rays. A backscatter detector can be used to detect these X-rays. Since the energy of the X-ray beam is material-specific, the element can be identified this way [46]. All scans were performed with 20 keV accelerating voltage.

Samples t1A and t1B were split. One part of each sample was analyzed on both sides in its original state, and the other part was etched with a mixture of concentrated hydrofluoric acid and concentrated sulfuric acid (mixing ratio 1:1) for 10 min and analyzed on both sides by SEM-EDX. In addition, another part of sample t1B was etched with concentrated hydrofluoric acid for one minute and analyzed by SEM-EDX. The SEM-EDX analysis was performed according to DIN ISO 22309.

### 2.2. Investigating Glass Composition ICP-OES

In order to investigate the exact composition of the glass, optical emission spectrometry with inductively coupled plasma (ICP-OES) was performed (ICP-OES Thermo Scientific iCAP 6300 Duo, Thermo Fischer Scientific Inc., Waltham, MA, USA). The samples were first dried and homogenized at 115 °C according to DIN 52331 with an oven (Universal oven UF260, Memmert GmbH and Co. KG, Schwabach, Germany). Subsequently, the samples were analyzed by ICP-OES, according to DIN 51086-2. In this method, the material is heated by an argon plasma, which has a temperature of about 10,000 K. The atoms in the vaporized sample are excited and emit material-specific electromagnetic radiation. This radiation can then be analyzed in a spectrometer [47].

### 2.3. Melting Experiment

Four melting tests were carried out. The adhesive film, which holds the DSSCs together, was removed manually from all DSSCs. To simulate a future glass recycling process, each test batch consisted of 40% raw material and 60% cullet (glass intended for a remelting process) from various sources. The raw material for glass production consists mainly of silica sand and in smaller quantities of lime, ash, dolomite and soda ash [48].

The composition of the raw material mixture used in this experiment is shown in Table 1.

The first melt was the reference sample and consisted of a typical soda-lime-white glass mixture (40%), as can be seen in Table 1 and white cullet (60%), hereafter referred to as Melt A. The cullet used was a typical white container glass from a German processing plant.

The second melt was also a typical soda-lime-white glass mixture, this time with the addition of DSSCs (with glycerol and poly (ethylene oxide) (PEO), which were previously built in 2020 and used as sample 1 in reference [45].

Regarding the applied simulated weathering process, the DSSCs of Melt B were washed with tap water and then dried in the oven at 115 °C (Universal oven UF260, Memmert GmbH and Co. KG, Schwabach, Germany).

The PEO served as a gel electrolyte in the DSSCs and helped to improve the long-term stability of the DSSC. For future applications, the use of gel or solid-state electrolytes could lead to unwanted plastic components in the melt, hereafter referred to as Melt B.

The third melt was a typical soda-lime-white glass mixture with addition of DSSCs, namely, sample 2 from reference [45], with PEO and without simulated weathering process, hereafter referred to as Melt C.

The fourth melt was a typical soda-lime-white glass mixture with addition of DSSCs from unpublished experiments conducted in 2018, without PEO and without simulated weathering process, hereafter referred to as Melt D.

The exact composition of the DSSCs of Melt B and C can be taken from reference [45]. Regarding Melt D, the DSSCs were also based on Man Solar glass slides and Mayfair-forest fruit tea (Mayfair, Wilken Tee GmbH, Fulda, Germany) as a natural dye, but a commercial liquid iodine-triiodide electrolyte purchased from Man Solar was used instead of a gel electrolyte. Graphite was applied as a catalyzer layer by pencil (grade 6B, purchased from J. S. Staedtler) and by graphite spray (Kontakt Chemie, Graphit 33). Their general design is similar to that of the DSSCs used in reference [8,11].

Table 2 shows the estimation of the DSSC cullet composition (a detailed explanation can be found in Section 3.5—Industrial Scale-Up and Volume Estimation).

Table 3 shows the composition of the gel electrolyte of the cells used for the different melts.

Table 4 finally gives the overview of what each melt consisted of.

The DSSCs were crushed into pieces with a diameter of 0.8 mm using a hammer mill (Mixer Mill MM 400, Retsch GmbH, Haan, Germany). Melting was performed at 1300 °C for about 24 h in a furnace (laboratory chamber furnace-CWF, Carbolite Gero GmbH & Co. KG, Hope, United Kingdom). During the melting phase and at the beginning of the melting process, the state of the melts was visually observed and compared.

Three lab spatula of the material were added to the melting pots. After 5 min in the furnace, the melts were visually evaluated and more material was added to the melting pots (3 more spoonfuls of batch material). This procedure was repeated 20 min after the start of the experiment and 50 min after the start of the experiment. After that, the melting process was continued for 24 h.

After the melting test, the melts cooled and were examined with a microscope (SteREO Discovery.V20, Zeiss AG, Oberkochen, Germany) according to ISO 8039.

## 3. Results and Discussion

### 3.1. SEM-EDX

**Figure 1:**
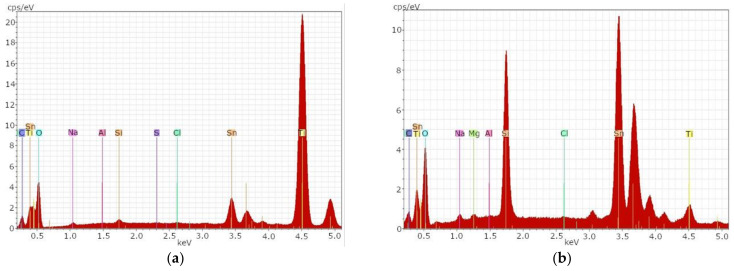
SEM-EDX result for sample t1A, the TiO_2_-coated front electrode of a DSSC: (**a**) before etching; (**b**) after etching.

Both electrodes are coated with FTO from one side, and the front electrode has an additional TiO_2_ layer. This could be confirmed with the SEM-EDX. For both samples, a surface with predominantly glass components could be detected. Figure 1a shows the EDX spectrum of sample t1A. While C, Sn, Ti, O, Na and Si, as well as Al, indicate the glass components in the deeper layers, Ti and Sn are the front layers indicating TiO_2_ and FTO.

**Figure 2:**
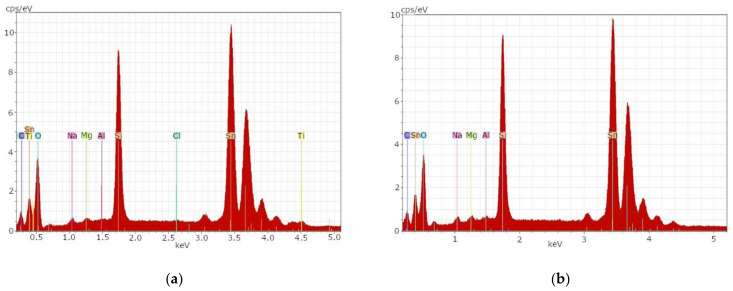
SEM-EDX result for sample t1B, the FTO-coated back electrode of a DSSC: (**a**) before etching; (**b**) after etching with hydrofluoric acid 1 min.

In Figure 1 and Figure 2, the *x*-axis shows the energy of the X-rays emitted by the electrons jumping to a lower energy level. In this way, conclusions can be drawn about the elements of the sample. The *y*-axis shows the number of photons per second and electron volt (cps/eV).

Very high concentrations of the element titanium and low concentrations of the element tin were detected on the surface of sample t1A. Typical glass components could only be detected in low concentrations.

Figure 1b shows the same sample after the etching process.

After the etching process, a significant decrease of the element titanium and a significant increase of the element tin could be determined. Elements typical for glass could be determined in significantly higher concentrations after the etching process. This indicates that the TiO_2_ layer on the glass surface can be removed with an etching process.

Figure 2a shows the EDX spectrum of sample t1B before the etching process. High concentrations of the element tin as well as elevated glass-typical elements could be determined. The glass-typical elements can be seen in the left part of Figure 2a. 

After the first 10 min etch with a mixture of hydrofluoric acid and sulfuric acid (1:1), sample t1B showed no differences from Figure 2a. Therefore, a second etch with concentrated hydrofluoric acid was subsequently performed for one minute. The result can be seen in Figure 2b. After both etching processes, an almost unchanged state of the glass surface with high concentrations of tin and elevated concentrations of glass-typical elements could be determined. Table 5 provides an overview of the elements detected in the SEM-EDX analysis.

Even after the etching processes, Sn could still be measured for both samples. This indicates incomplete removal of the layers present on the glass surface. However, TiO_2_ can be removed with acid. This suggests that valuable FTO-coated glass or TiO_2_ could be recovered through a chemical recycling process. Rather than recycling the glass, the FTO-coated glasses could be used in a remanufacturing process for new DSSCs [49]. The FTO layer is resilient and cannot be removed by the described etching process. If the FTO layer needs to be removed, e.g., for a possible recycling process, sulfuric acid or hydroiodic acid is required [50,51].

### 3.2. ICP-OES

In Table 6. The results of the ICP-EOS are shown. In the right column, values of a patent glass especially for photovoltaic usage are shown for comparison. Significant differences are in the values of Al_2_O_3_. Sample t1A has significantly higher values than t1B. The patent glass recommends even higher values and shows typical refining agents such as sulfates, chloride, Sb_2_O_3_, As_2_O_3_ and SnO_2_ [52]. Aluminum oxide can increase the viscosity of the glass as well as its chemical resistance [53]. CaO is also used in glass production to increase viscosity and improve chemical resistance [54]. Therefore, it is possible that either AlO_3_ or CaO is used in production due to price advantages of the materials.

To reach highest efficiencies in photovoltaic applications, the Fe_2_O_3_ part in the glass should be as small as possible because the light transmission through the glass is reduced by iron. Fe_2_O_3_ absorbs photons of the UV spectrum, which reduces the efficiency of the module [52]. For c-Si PV, even small amounts of Fe_2_O_3_, such as 1% in the front glass, reduce the efficiency by about 9.8% [55]. The glass thickness also influences the transmission rate (thin glasses usually have higher transmission values) [56].

However, maximizing transmission and thus efficiency shortens the lifetime of the entire c-Si PV system for conventional photovoltaic applications, which are usually laminated with ethylene-vinyl acetate (EVA) foil. The UV light is responsible for degradation of the foil and semiconductor material, which leads to efficiency losses or failure of the module [57]. Therefore it is necessary to balance photovoltaic efficiency and degradation by incorporating the right amount of Fe_2_O_3_ into the cover glass [56]. This is also important for DSSCs due to aging processes inflicted by thermal stress and UV light [58]. For a gel electrolyte DSSCs, thermal stress is less relevant because it exhibits higher thermal- and photostability, compared to liquid electrolyte DSSCs [59]. An optimal cover glass for DSSCs must be matched to the dye used and the specific absorption spectrum.

In the case of anthocyanin, the absorption spectrum has maxima at 465–560 nm and 265–275 nm [60]. Light with a wavelength of more than 560 nm would only heat up the DSSCs’ thermal stress. Light in the ultraviolet range would lead to faster degradation of the DSSC. Therefore, it is a balancing act between more efficiency and higher life expectancy of the DSSCs. A cover glass that reflects or filters unnecessary wavelengths could improve longevity without reducing the DSSCs’ efficiency. However, the absorption spectrum of anthocyanins can be modified by adjusting the pH of the dye [61]. The efficiency increases at lower pH values. Junger et al. lowered the pH value from 2.3 to 1.1 and observed that the efficiency could be doubled in this way [62]. In addition, copigmentation with caffeine can improve the efficiency of anthocyanin-based DSSCs and the original pH (2.3) can be maintained as the range of maximum efficiency is shifted from lower (1.1) to higher (2.3) pH values [63]. Furthermore, a bathochromic shift of the anthocyanin spectrum can be observed when the dye is applied to TiO_2_. The absorption maximum of the spectrum then shifts to higher wavelengths and lower light intensities [8]. This indicates that the appropriate adjustment of cover glass and dye absorption spectrum has several configuration options. In addition, different dyes are combined to improve the absorption spectra and thus the energy conversion efficiency. For example, Bashar et al. combined dyes from beetroot (80%) and spinach (20%) and significantly improved the energy conversion efficiency from 0.56% (beetroot) and 0.49% (spinach) to 0.99% (combination of dyes) [64]. This shows that, in addition to matching the cover glass to the absorption spectra, co-sensitized DSSCs can be built from a variety of dyes to improve their power-conversion efficiency.

### 3.3. Melting Experiment

**Figure 3:**
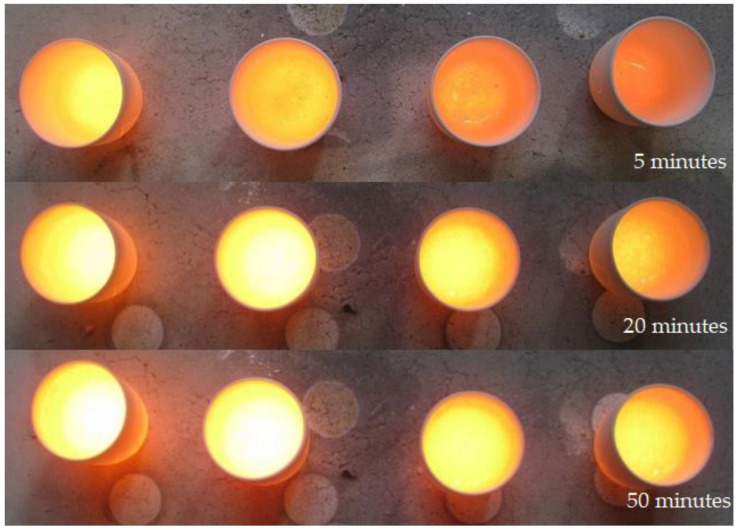
Glass melting after approx. 5, 20 and 50 min after the start of the melting process. From left to right A, B, C and D.

Figure 3 shows the melts about 5, 20 and 50 min after the start of the melting process. After 5 min, melts A and B already showed quite a lot of molten glass with seeds (air inclusions < 1 mm) and bubbles (air inclusions > 1 mm). Melts C and D, on the other hand, still exhibited significantly more unmelted batch and larger bubbles. Melt D, however, was located at the front of the furnace and was therefore introduced into the furnace last and removed first. The unmelted batch can be explained by the shortest melting time.

After 20 min, seeds and unmelted components could be determined. In direct comparison, melt D had the highest proportion of unmelted components and melt A the highest proportion of seeds.

**Figure 4:**
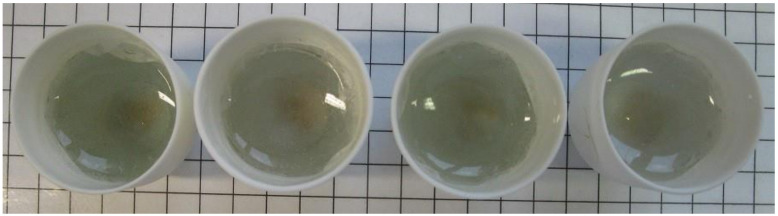
Melts after completion and cool down of the melting process. From left to right A, B, C and D.

Approximately 50 min after the start of the melting process no unmelted components could be detected. Melt A and B showed optically coarser bubbles than melt C and D. Figure 4 shows the melts after completion of the experiment and after the samples had reached room temperature. All four melts show pinkish streaks at the bottom. Since the control sample (melt A) also shows the pinkish streaks, they have to be caused by impurities that do not originate from the DSSCs. Visually, the melts exhibited slightly different colorations. Melt A showed a slightly bluish coloration, melt C showed a slightly greenish coloration, and melt D showed a slightly pink coloration. The bluish coloration of melt A might originate from higher Fe_2_O_3_ concentrations in the glass [54]. Melt B was very clear and without any clearly discernible coloration. The colorations of melt C and D could originate from contaminations such as dust, fingerprints and so on. This indicates that a weathering process with simple water could increase the quality of the melt.

### 3.4. Microscopic Examination of the Glass

**Figure 5:**
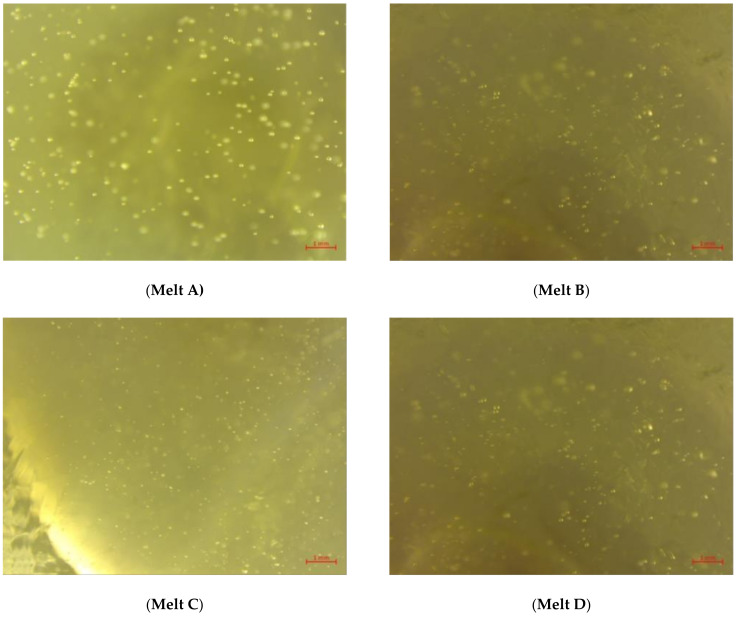
Microscopic images of the melts **A**–**D**.

For further examination of the glass quality, the samples were examined with a microscope. The results can be seen in Figure 5.

No inclusions (foreign bodies), such as small metal or ceramic pieces, were found in any of the melts. The melts were visually similar. Melt C had the most streaks and the most, predominantly small, seeds. Slightly fewer streaks were visible in melt D than in melt C. In addition, the seeds present in the melt were somewhat larger. Melt A also had slightly larger seeds and fewer detectable streaks than melt D. The most seeds were found in melt B. The seeds were also slightly larger than in melt C. Melt B had the fewest discernible streaks. The melts with DSSC cullet are of similar quality compared to the reference sample. These results indicate that DSSCs, as described in this paper, are suitable for a glass recycling process. Further investigations need to prove that the quality of DSSC recycling glass is sufficient for container or flat glass. Gases that may have been generated during the melting process were not monitored and should be investigated, especially if plastic material contaminates the melt (PEO in this case). During the melting process, PEO is thermally decomposed. This process takes place in a temperature range from 324 °C to 363 °C [65].

### 3.5. Industrial Scale-Up and Volume Estimation

The DSSC market is growing [7]. For this reason, an increasing amount of DSSC materials will require proper recycling processes in the future. The previous melting experiment shows that at least non-toxic glass-based DSSCs have high potential for glass recycling. To better understand the amount and composition of material that could enter recycling facilities, it is estimated what a ton of DSSC material consists of. For recycling plants, it is necessary to know the composition of the batch in order to properly adjust the recycling processes.

Table 7 shows an estimate of how much material of what type could accrue in recycling facilities in the future due to DSSC waste. The first column shows the material for our non-toxic DSSCs. Column two shows an estimate from Parisi et al., who did a life cycle assessment for DSSCs [41]. The glass thickness and the side lengths were determined with a caliper gauge. The diameter of the glass plates is 7 cm^2^, and the thickness is 0.1 cm. The TiO_2_ layer is about 6–10 μm thick and is applied to about 6 cm^2^. The FTO layer is very thin and invisible; in this case, we estimated that the layer is about 10 nm thick and is applied to the entire surface of the glass, i.e., 7 cm^2^ each for the front and counter electrode. Two drops of the commercial liquid electrolyte are used for a typical DSSC without PEO. Each drop has a volume of about 20–25 μL. Although the electrolyte evaporates over time and not much of it would remain in a final recycling process, we listed it because residual chemicals could still interfere with the recycling process. We have estimated a thickness of 2 µm for the graphite layer. During the dyeing process, a monomolecular dye layer adheres to the TiO_2_ layer. After the useful life of a DSSC, the organic dye is decomposed by light or completely degraded in a final composting process before recycling. No gel electrolyte or sealing was considered for the basic DSSCs.

The following material densities were used for the calculation of the material mass:

- Glass: 2.5 g/cm^3^ [66],
- TiO_2_ 4.23 g/cm^3^ [67],
- FTO/SnO_2_ 6.99 g/cm^3^ [68] for thin films; the density of FTO is close to the density of SnO2 [69],
- Electrolyte: iodine/potassium iodide 1.12 g/cm^3^ [70],
- Graphite 2.26 g/cm^3^ [71].

The volumes were multiplied by the densities and scaled up to one ton of material. The results can be seen in Table 7.

Both estimates are similar to each other. According to our estimate, the electrolyte volume is more than twice as large. More efficient production processes could probably reduce this amount. In the estimate of Parisi et al. a toxic ruthenium dye is used. This material hinders the recycling process and would poison the material cycle. Therefore, such modules require an additional recycling step in which the toxic dye is separated from the glass material [42]. DSSCs with organic dyes, on the other hand, could be used directly in glass recycling after a natural weathering process.

TiO_2_ is commonly used for the construction of DSSCs and represents the best compromise between sustainability and high efficiency [72]. This material is quite safe and abundant [73,74]. In France, however, TiO_2_ has been banned from foods because studies suggest that TiO_2_ nanoparticles may cause health problems in rats [75]. Due to its white color, TiO_2_ is often used in food as food additive E 171. TiO_2_ is already used in a variety of applications, especially in the construction sector [76]. TiO_2_ is, moreover, already a component of glass and could be used in glass recycling. However, recovery in a separation process before recycling could be advantageous, e.g., reusing it directly for the production of new DSSCs.

No platinum, silver or thermoplastic is used in our basic DSSCs. In the future, however, foils or films to prevent glass breakage or metal elements to improve conductivity may be required. However, there are also metal-free solutions, such as carbon nanotubes, to improve conductivity [15]. Thermoplastics can be used as sealants to prevent electrolyte leakage, solvent evaporation and electrolyte corrosion [77]. As applied with the thermoplastic PEO to the DSSCs in this manuscript, melt C and D.

Since more and more photovoltaic modules already have to be recycled, there is experience of what problems arise in the process. This knowledge can be used to develop more sustainable DSSCs. DSSCs have some components, such as the glass or a protective film, that are also found in current c-Si PV modules [44].

**Figure 6:**
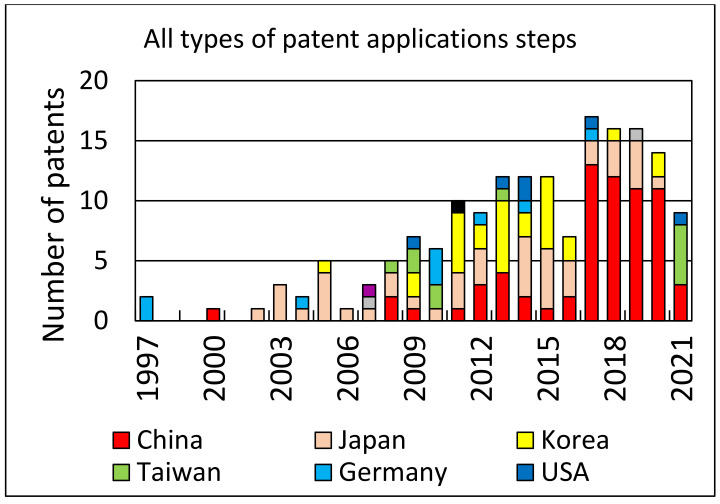
Distribution of the number of patents by country (all steps of a patent application process including the first step A): China—67, Japan—44, Korea—29, Taiwan—11, Germany—9, USA—6, India—2, Great Britain—1 and Switzerland—1.

**Figure 7:**
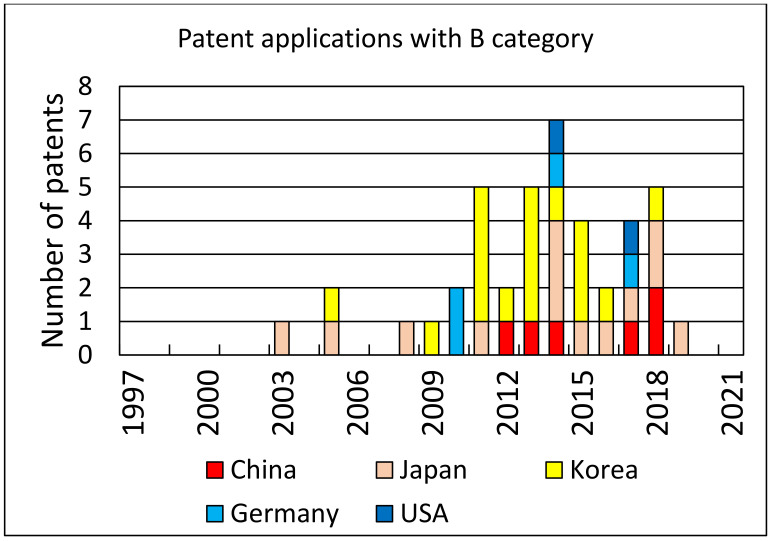
Distribution of the number of patents by country (the B step of a patent application process meaning at least publication and preliminary approval): Korea—17, Japan—13, China—6, Germany—4 and USA—1.

An analysis of patent applications and the number of patents granted over the years can provide some measure of the interest in the topic of photovoltaic cell recycling. Using Web of Science resources and the Derwent Innovations Index database, where we searched patent titles with the keywords “solar cell recycling”, we found 170 patents with all categories of patent approval levels. For approved patents (at least category B), we found 25 cases. Both search results are shown in the form of diagrams in Figure 6 and Figure 7, respectively.

The predominant technological recycling methods found in the descriptions of the patents are as follows: removal of electrodes from solar cells, separation of anti-reflective layers, recycling of electronic chips, reuse of electrically conductive glass substrates, recovery of atomic elements including silver and indium, removal of aluminum layers, melting of tempered glass material, and recycling of crystalline silicon, among others.

Overall, it has not yet been possible to recover high-quality materials from the c-SI PV module using a conventional glass recycling process [78]. Due to the shredding of the modules, the materials are mixed and are difficult to separate by type.

In c-Si PV, EVA foil complicates the recycling process. One solution could be the use of polyvinyl butyral (PVB) foil, which is also used in safety glass and for which mechanical and chemical recycling processes exist [79,80]. There is also a patent that describes a method of separating the laminated film from the glass [81]. This patent is not for c-Si PV but for laminated safety glass. However, it could also be a model for recycling c-Si PV modules using a similar process. An example of a photovoltaic application with PVB film is Trosifol Solar [82]. They already use PVB film in their photovoltaic modules. For recycling reasons, we recommend the use of PVB in future DSSC applications, if a film has to be used at all.

The metal elements platinum and silver hinder the glass recycling process. So far, the high-grade, low-iron glass from c-Si PV modules can only be used in a downcycling process because during the recycling process the cullet is contaminated with iron parts [78].

In addition, there is the contamination by film residues and the silicon wafers. The EOL-Cycle research project shows that an optimized recycling concept with more complex and numerous cleaning and sorting steps enables the recovery of high-quality materials. However, it was not yet possible to achieve a sufficient degree of purity for the production of flat glass [83]. Furthermore, there is the additional technical effort, which has a negative impact on the economic efficiency of the process. Other problems with c-SI PV recycling are the fluctuating composition of the PV modules and the use of materials that are not pure, such as the use of different polymers [83].

A company from Japan is successfully using an automated system in which the glass is separated from other components with a highly heated knife [84]. With such advanced recycling processes, a downcycling process, at least for the glass, can be avoided.

By learning from the difficulties of recycling conventional PV technologies, DSSCs can be made more suitable for future recycling. At least 70% of the environmental impact of a product is determined in the design phase of the product. Therefore, it is reasonable to invest effort into reducing the environmental impact of a product already in the design phase [85]. By using recycled material or closing material loops for DSSCs, the energy requirement for production can be reduced. The production of TCO glass in particular is energy-intensive, and it would be beneficial to reuse the glass for constructing new DSSCs [86]. Considering the global climate crisis and resource scarcity, sustainability should be given the same importance as efficiency and stability in DSSC research [13,44]. In the development of DSSC for the mass market, recycling must already be taken into account in the design phase.

## 4. Conclusions

The experiment shows that the TiO_2_ could possibly be separated from the front electrode by wet chemical processes. However, the FTO layer is very robust and cannot be detached by the acid used.

Chemical analyses by ICP-OES revealed significant differences in the elements of the two base glasses studied. In particular, significant differences were found for the elements aluminum oxide and iron oxide.

The melting tests carried out showed slight differences in the melting behavior. In the molten glass, the melts were visually quite similar but had slight color differences. Melt B exhibited the highest brilliance and the lowest visually perceptible color impression, while melt D had a slightly pinkish tint and melt C a slightly greenish tint. All melts exhibited visually perceptible color impression and showed a pinkish streak at the bottom of the melt.

None of the samples showed inclusions in the glass melt, but visually perceptible seeds could be detected. In general, all melts were similar. However, melt B had the fewest seeds and visually perceptible streaks. In direct comparison, there were no distinctive differences from the reference sample in either the melting behavior of the DSSC mixtures or the glasses resulting from the melts. Further tests are necessary to investigate the suitability of the resulting material for float and container glass production. For example, material properties such as chemical resistance, transparency or viscosity must be evaluated.

## Figures and Tables

**Table 1:** Composition of the used batch in weight percentage.

Sand	Soda	Dolomite	Nepheline	Calumite *	Lime	Sodium Sulfate
59.2%	17.2%	12.4%	5.0%	2.5%	2.5%	1.2%

Glassy calcium aluminum silicate produced from granulated blast furnace slag.

**Table 2:** Estimated composition of the DSSC cullet in weight percentage.

FTO Glass	TiO_2_	Electrolyte	Graphite
97.7%	0.7%	1.5%	0.1%

**Table 3:** Composition of the gel electrolyte of DSSCs used for the melting tests [45] in weight percentage.

Melt	600 kg/mol PEO	Glycerol
A (no electrolyte)	none	none
B	17%	38%
C	8%	47%
D (iodine/potassium iodide electrolyte)	none	none

**Table 4:** Overview of the composition of each melt.

Melt	Composition
A	60% white cullet + 40% batch (Table 1)
B	60% DSSC cullet (Table 2) and electrolyte with PEO (Table 3/B) + 40% batch (Table 1)
C	60% DSSC cullet (Table 2) and electrolyte with PEO (Table 3/C) + 40% batch (Table 1)
D	60% DSSC cullet (Table 2) and electrolyte without PEO (Table 3/D) + 40% batch (Table 1)

**Table 5:** Overview of the elements detected with SEM-EDX.

Sample	Detected Elements									
t1A before etching (Figure 1a)	Ti	Sn	Cl	Si	Al		Na	O	C	S
t1A after etching (Figure 1b)	Ti	Sn	Cl	Si	Al	Mg	Na	O	C	
t1B before etching (Figure 2a)	Ti	Sn	Cl	Si	Al	Mg	Na	O	C	
t1B after etching (Figure 2b)		Sn		Si	Al	Mg	Na	O	C	

**Table 6:** Chemical analysis by ICP-EOS, results in weight percentage.

Element	t1A (%)	t1B (%)	Patent Glass [52] (%)
Al_2_O_3_	0.54	0.07	4.7–19
Fe_2_O_3_	0.009	0.100	0–0.5
CaO	8.95	8.88	0–5
MgO	4.26	3.96	0–6
SrO	0.005	0.006	0–7
Na_2_O	13.80	13.64	10–18
K_2_O	0.05	0.04	0–8
Li_2_O	0.003	0.002	0–4
BaO	0.001	0.001	0–10
PbO	0	0.0001	–
TiO_2_	0.005	0.010	0–6
Cr_2_O_3_	0.0003	0.0006	–
Mn_2_O_3_	0.001	0.006	–
NiO	0.0005	0.0003	–
SnO_2_	0.013	0.024	–
ZnO	0.002	0.003	0-0.3
ZrO_2_	0.00	0.01	0–0.5
SO_3_	0.220	0.218	–
SiO_2_	72.14	73.03	49–69

**Table 7:** Material composition of one ton of DSSC.

Material	Mass for 1 t of DSSCs, Basic Cells	Material	Mass for 1 t of DSSCs, Parisi et al. [41]
FTO glass	977 kg	TCO glass	955 kg
TiO_2_	7 kg	TiO_2_	5 kg
Electrolyte	15 kg	I /I_3_ liquid solution	6 kg
Graphite	1 kg	Platinum paste	1.5 kg
Dye, anthocyanin *	-	Dye, Z907	0.06 kg
-	-	Silver paste	1 kg
-	-	Thermoplastic	31 kg

* The front electrode is bathed in a solution of 2.5 g tea, 22.5 g of deionized water and 7.5 g of ethanol. Several DSSCs can be dyed within one procedure. The biodegradable dye is not worth to be recycled in contrast to the ruthenium dyes [8].

## Data Availability

Not applicable.
